# Ocular surface microbiota: Ophthalmic infectious disease and probiotics

**DOI:** 10.3389/fmicb.2022.952473

**Published:** 2022-08-19

**Authors:** Ming-Cheng Chiang, Edward Chern

**Affiliations:** ^1^niChe Lab for Stem Cell and Regenerative Medicine, Department of Biochemical Science and Technology, National Taiwan University, Taipei, Taiwan; ^2^Research Center for Developmental Biology and Regenerative Medicine, National Taiwan University, Taipei, Taiwan

**Keywords:** ocular surface microbiota, ophthalmic infectious disease, gut–eye axis, probiotic, microbiome

## Abstract

Recently, increasing studies have emphasized the importance of commensal bacteria in humans, including microbiota in the oral cavity, gut, vagina, or skin. Ocular surface microbiota (OSM) is gaining great importance as new methodologies for bacteria DNA sequencing have been published. This review outlines the current understanding and investigation of OSM and introduces the new concept of the gut–eye axis. Moreover, we have collected current studies that focus on the relationship between ophthalmic infectious disease and alterations in the OSM or human gut microbiota. Finally, we discuss the current application of probiotics in ophthalmic infectious disease, its limitations to date, and futural directions.

## Introduction

Microbiota is a collective term for polymicrobial communities, including bacteria, virus, fungi, and archaea. These may inhabit a particular site, shape the microenvironment, and play a role in metabolism, disease development, and immunomodulation in the human body (De Sordi et al., 2019). Microbiota in the gastrointestinal tract is well recognized to modulate homeostasis of the digestive system. Its clinical applications are numerous; however, the ocular surface has its own microbiota that is related to several ophthalmic diseases (Zegans and Van Gelder, 2014). Moreover, OSM can inhibit the growth of pathogens and is highly related to eye infections. OSM is composed of several microorganisms, ranging from bacteria to fungus. The variations in OSM can be caused by personal habits, systemic diseases, or antibiotic usage, leading to disturbances in the local immune function. Moreover, the gut–eye axis, which illustrates the relationship between gastrointestinal microbiota and the ocular microenvironment, has been proposed. Combined with better sequencing skill and furthering understanding of human microbiota, the application of probiotics to improve ocular or gastrointestinal microbiota and for ophthalmic infectious disease treatment is an important object of study.

## Properties of OSM and OSM identification

### Local immune system and microbiota

The ocular surface is a defensive frontline of the innate immune system. It is composed of the cornea and sclera, and their overlying tissue, such as the conjunctiva and tear film. It forms a physical barrier through the presence of epithelial intercellular tight junctions (Mantelli et al., 2013). The tear film contains several anti-infectious molecules, including lysozymes, lactoferrin, lipocalin, and β-defensins (McDermott, 2013). Resident conjunctiva lymphocytes, plasma cells, macrophages, and dendritic cells (DC) can be triggered to produce antibodies and upregulate the downstream immune response, eliminating non-self microorganisms by phagocytosis and intracellular degradation (Bauer et al., 2002; Petrillo et al., 2020). The ocular surface is armored with various antimicrobial strategies; however, it can tolerate some commensal colonies, such as OSM. Lipopolysaccharides (LPSs) are the major component of the outer membrane of Gram-negative bacteria (GNB), which serves as a barrier resistant to host-derived antimicrobial compounds (Erridge et al., 2002). LPSs are pathogen-associated molecular patterns (PAMPs). This means that they can be recognized as a molecular hallmark of invading microbes by the host (Erridge, 2010). In addition, lipoteichoic acid in Gram-positive bacteria (GPB), lipoproteins, and mycobacterial lipoglycans also meet the criteria of PAMPs (Ray et al., 2013). Pattern recognition receptors (PRRs) are surface proteins of antigen-presenting cells (APCs) capable of recognizing PAMPs (Gordon, 2002). Once PRRs recognize and link to the PAMP, a downstream signaling process is initiated and production of pro-inflammatory cytokines are upregulated as the PRR–PAMP pair is an invasive signal for the immune system (Mogensen, 2009). However, PRRs contain various subfamilies. Toll-like receptors (TLRs), most widely known among all, are closely related to the immune silence of the ocular surface (Takeuchi and Akira, 2010).

#### TLRs

TLRs are transmembrane receptors, which can be divided into two groups depending on their cellular localization and PAMP ligands: One group comprises TLR1, TLR2, TLR4, TLR5, TLR6, and TLR11, which are categorized together as they are located on cell surfaces and recognize microbial membrane components; the other group is composed of TLR3, TLR7, TLR8, and TLR9, which are located in intracellular vesicles and recognize microbial nucleic acids. Each TLR has its own specific characteristic and function. TLR2 senses lipoproteins by forming a heterodimer with either TLR1 or TLR6. TLR4 combined with myeloid differentiation factor 2 appears to recognize LPSs. Hayashi et al. (2001) found bacterial flagellin, a binding ligand of TLR5. TLR11 recognizes the profilin-like molecule derived from *Toxoplasma gondii*; however, the TLR11 functional protein does not exist in humans because a stop codon is contained in its open reading frame. TLR3 can identify the double-stranded RNA of some viruses, while TLR7 and TLR8 recognize single-stranded RNA. TLR9 can be activated by microbial DNA sequences containing unmethylated CpG dinucleotides. After binding with specific ligands and being activated, TLRs trigger downstream signal pathways and inflammatory gene transcription, contributing to antimicrobial response.

#### Immune silence

A complicated defensive system inhibits microbial growth on the ocular surface; however, some microorganisms are tolerable to the human immune system. Immune system homeostasis is maintained by the balance between regulatory and stimulative signals as antigen-loaded DCs migrate to lymph nodes and induce regulatory or immunogenic T cells (Horwitz et al., 2019). The type of T-cell expansion that is produced is determined by the presence of danger signals, such as PAMPs or endogenous signals derived from cell damage (Idzko et al., 2007). By contrast, an unclear mechanism imprints the tolerogenic profile on ocular surface DCs in basal conditions. Once DCs loaded with ocular surface-derived antigens contact circulating naive T cells, regulatory T cells are induced because tolerogenic profiles have been imprinted on DCs previously (Galletti et al., 2017). Several hypotheses have been published explaining the immune silence phenomenon on the ocular surface. TLR2 and TLR4 are expressed intracellularly in human corneal epithelial cells (Ueta et al., 2004). TLR5 is located at wing cells and the basal cell layer, instead of the outermost part of the cornea; therefore, bacteria cannot bind to TLR5 as long as the integrity of the epithelial barrier persists (Zhang et al., 2003). Thus, immune activity at the ocular surface is regulated by various mechanisms, leading to an immune-silent environment within which microbiota can survive.

### Members of ocular microbiota

#### Culture-dependent method

The ocular surface is constantly exposed to the environment. Whether the stable presence of commensal bacteria exists remains under debate. In the past, OSM was harvested from tears or swabs of the ocular surface and cultured. Of all cultivable microorganisms from conjunctiva swabs, GPB, including *Staphylococcus, Propionibacterium* sp., diphtheroids, and *Staphylococcus aureus*, accounted for the majority. In addition, GNB, such as *Pseudomonas* sp., *Enterobacter* sp., and *Escherichia coli*, and fungus were found in healthy eyes, although less common. Overall, coagulase-negative staphylococci were the most common bacteria isolated from the conjunctiva, lids, or tears (Gordon, 2002). However, traditional culture methods for OSM identification are seldom used currently due to their limited detection of slow growing, fastidious, or uncultivable species.

#### Culture-independent method

Next-generation 16S rRNA sequencing has been applied to microorganism identification over time. rRNA is the most conserved and least variable gene in all cells, belonging to the same genus and species. 16S rRNA is a small ribosomal subunit containing 1,500 nucleotides, composed of hypervariable regions and strongly conserved regions. A primer is designed to bind conserved regions and amplify the variable parts between different genera and species. Thus, 16S rRNA sequencing is a tool to classify bacteria to the species level and recognize closely related bacterial species. As a result, this sequencing has enabled the classification of uncultivable bacteria and the tracing of phylogenetic relationships, leading to tremendous development in taxonomy.

#### Application to OSM

16S rRNA sequencing has been applied to OSM identification. Graham et al. identified the bacterial genera of conjunctiva samples from 91 subjects by culture and 16S rRNA sequencing. Much more genera could be identified by 16S rRNA sequencing than by the culture method. It revealed coagulase-negative *Staphylococcus* sp., *Staphylococcus epidermidis, Bacillus* sp., *Rhodococcus* sp., and uncultivable bacterium, including *Corynebacterium* sp., *Klebsiella* sp., and *Erwinia* sp. By contrast, only coagulase-negative *Staphylococcus* and *Bacillus* sp. were grown in culture (Graham et al., 2007). In addition, fungi were identified as a member of OSM. A total of two phyla, *Basidiomycota* and *Ascomycota*, and five genera, *Malassezia, Rhodotorula, Davidiella, Aspergillus*, and *Alternaria*, were found to account for >80% of fungal flora and were isolated from >80% of 45 tested samples (Wang et al., 2020). Viruses are found on the ocular surface. A study has demonstrated the presence of torque teno virus on the ocular surface in 65% of 107 healthy volunteers (Doan et al., 2016).

In microbial ecology, the term “microbiome” is an assembly of the genomes of microbial symbionts that live inside and on humans, and “core microbiome” is defined as the set of genes present in a given habitat in all or the vast majority of humans (Turnbaugh et al., 2007). However, there is a lack of consensus about whether core microbiomes exist on the ocular surface. Dong et al. utilized 16S rRNA sequencing to survey OSM of both eyes in four subjects. A total of 12 genera—*Pseudomonas, Propionibacterium, Bradyrhizobium, Corynebacterium, Acinetobacter, Brevundimonas, Staphylococcus, Aquabacterium, Sphingomonas, Streptococcus, Streptophyta*, and *Methylobacterium*—are considered the putative core microbiomes as they were ubiquitous among all examined subjects (Dong et al., 2011). Ozkan et al. sampled the conjunctiva of 43 healthy subjects at three time points: baseline, 1 month, and 3 months. A total of 183 operational taxonomic units (OTUs) were collected after contamination filtering. Each subject carried 33 OTUs on average across the three time points. OTUs associated with *Corynebacterium, Streptococcus, Anaerococcus, Sphingomonas*, and *Acinetobacter* were found in one or more individuals throughout the whole study; however, no OTU was noted in all individuals at all time points or in all individuals in any one time point, indicating that the ocular surface may not carry consistent OTUs (Ozkan et al., 2017). This contrasts with the signature core microbiome of OTUs that are found on the human skin, in the gut, or in the oral cavity (Zaura et al., 2009; Zhu et al., 2010; Byrd et al., 2018). As a result, whether “core” residential microbiomes exist on the ocular surface remains unclear.

### Influence parameters of OSM and current limitations in OSM identification

#### Inherent factors

A study of 106 infants in which conjunctival swabs were taken immediately after birth showed that infants delivered vaginally carried more bacterial species and colony-forming units than infants delivered *via* cesarean delivery (Isenberg et al., 1988). The Shannon index is used to reflect the number of species or types in a database. The higher the number, the higher the species diversity. This was believed to be lower in the elderly (60–70 years old) than in younger adults (25–35 years old) (Suzuki et al., 2020). However, the opposite outcome has been published by Wen et al. as they found a higher Shannon index in an elderly group (Wen et al., 2017). These outcomes may be led by different sequencing technologies, for example, 16S rRNA sequencing and metagenomic shotgun sequencing. Previous studies have shown mixed opinions on the relationship between gender and OSM (Ozkan et al., 2017; Cavuoto et al., 2018), with no consensus reached to date.

**Figure 1:**
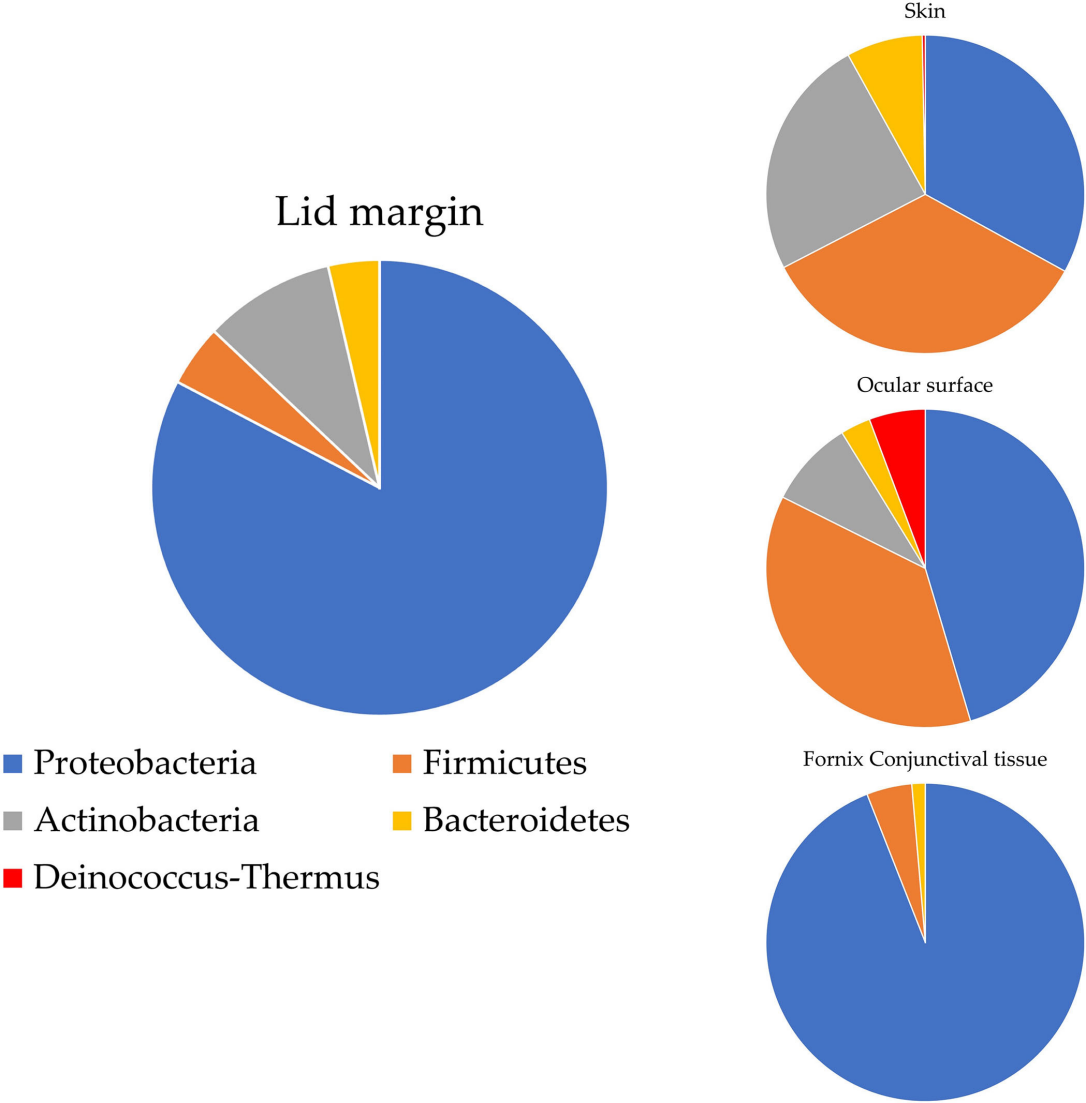
OSM distribution on ocular surface by Ozkan et al. (2019). *Proteobacteria* account for most of the ocular flora in most areas, while the periocular skin flora is predominated by *Firmicutes* and colonized with *Actinobacteria* more frequently than other areas.

**Figure 2:**
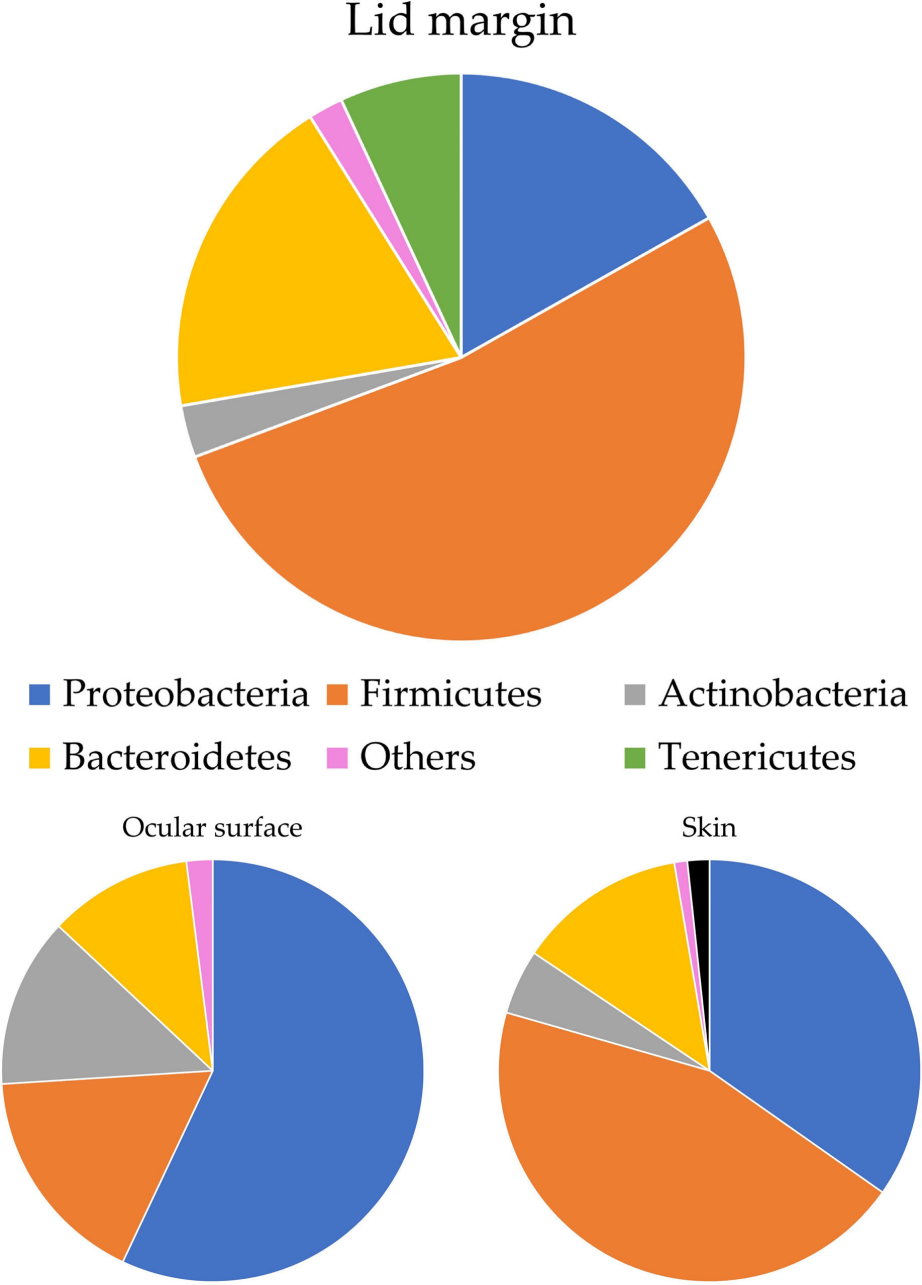
OSM distribution on ocular surface by Cavuoto et al. (2019). *Firmicutes* are the most abundant flora of the lid margin, while Ozkan et al. (2019) have reported that *Proteobacteria* is the most prevalent here. The two studies agree that *Proteobacteria* is the most common phyla of the ocular surface and that periocular skin is dominated by *Firmicutes*.

Ozkan et al. studied the bacterial biogeography in the human eyes and divided it into four areas: ocular surface, fornix conjunctiva tissue, lid margin, and skin 1 cm above the eyelid margin. Pseudomonas was often found on the fornix conjunctiva and lid margin, while *Acinetobacter* and *Aeribacillus* tended to be noted on the ocular surface. At the phylum level, *Proteobacteria* made up 90% of the OSM of the fornix conjunctival tissue, while *Bacteroidetes* appeared irregularly across all sites. *Actinobacteria* was more frequently found on the periocular skin than in other groups (Ozkan et al., 2019) (Figure 1). Detailed information at the genus level is shown in Table 1. Cavuoto et al. (2019) found that *Firmicutes* accounted for the majority of microbiota on the lid margin. This result differs from that of Ozkan et al. (2019); however, both found that *Proteobacteria* was predominant on the ocular surface, followed by *Firmicutes* and that *Firmicutes* had a slim lead over *Proteobacteria* on the periocular skin (Figure 2). A depth-stratified microbiota was identified by using samples collected from the same individual with different swab pressures. A greater relative abundance of *Staphylococcus* and *Corynebacterium* was noted with soft pressure swabs; the presence of *Proteobacteria* increased in swabs applied with firm pressure (Dong et al., 2011). Moreover, differences in OSM can exist between each eye in a same person (Zilliox et al., 2020).

**Table 1:** Members of OSM on ocular surface by Ozkan et al. (2019).

	**Bacterial phyla**	**Skin (%)**	**Lid margin (%)**	**Ocular surface (%)**	**Fornix conjunctiva (%)**
	*Proteobacteria*	31.6	77.4	44.6	90
	*Firmicutes*	32.9	4.1	36.3	4.4
	*Actinobacteria*	23.5	8.7	8.7	0
	*Bacteroidetes*	7.4	3.4	3	1.3
	*Deinococcus-Thermus*	0.3	0	5.6	0
	**Bacterial genera**	**Skin (%)**	**Lid margin (%)**	**Ocular surface (%)**	**Fornix conjunctiva (%)**
*Proteobacteria*	*Acetobacter*	0	0	6.9	0
	*Neisseriaceae*	9.6	5.1	6.8	1.5
	*Acinetobacter*	1	0.5	12.3	3.9
	*Sphingomonas*	0.3	0.1	2.5	0.4
	*Pseudomonas*	5.8	65.1	2.1	80.7
*Firmicutes*	*Staphylococcus*	15.2	1.8	3	1
	*Aeribacillus*	0.3	0	11.3	2
	*Streptococcus*	6	0.3	2.3	0
	*Bacillus*	0.4	0.2	5.9	0.3
	*Veillonella*	5.1	0	1.6	0
	*Thermoanaerobacterium*	0	0	4.5	0.3
	*Geobacillus*	0	0	2.8	0.5
*Actinobacteria*	*Corynebacterium*	12.2	7.8	4	0
*Deinococcus-Thermus*	*Deinococcus*	0.1	0	3.8	0

Only bacterial taxa with an average relative abundance >1% would be presented. F, family.

#### Acquired factors

A previous study pointed out that the OSM of newborns delivered vaginally carried more bacterial species and colony-forming units (Isenberg et al., 1988). However, conjunctival cultures collected 2 days after birth showed a more frequently positive outcome than those collected at birth, regardless of the delivery type, suggesting further shaping and modulation of OSM by diet and environment (Lee et al., 1989).

Antibiotics have been used for a long time to treat bacterial infections. However, antibiotic usage, either locally or systemically, can impact OSM. Dave et al. delivered azithromycin and fluoroquinolone intravitreally and then observed a significant change in ocular flora. *Staphylococcus epidermidis* and *Staphylococcus aureus* accounted for 54.5 and 18.2% of cultured isolates at baseline, respectively, which changed to 90.9% (*P* < 0.05) and 4.5% (*P* < 0.05) after azithromycin treatment. In the fluoroquinolone group, *Staphylococcus epidermidis* increased from 45.7 to 63.4% (*P* < 0.05) (Dave et al., 2013). Patients who underwent topical levofloxacin (LVFX) treatment for 1 month were recruited by Ono et al. to investigate the impact of antibiotics on OSM. The outcome showed substantially reduced bacterial diversity after LVFX usage (*P* < 0.01) (Ono et al., 2017). Moxifloxacin, a common topical-use antibiotic, is found to reduce culture positivity rates on the ocular surface (Celebi and Onerci Celebi, 2021). In addition, antibiotics do affect the drug resistance of microbiota as topical LVFX and moxifloxacin have been found to increase the mean MIC of ocular flora (Yin et al., 2013; Ono et al., 2017). Moreover, preoperative conjunctival isolates of coagulase-negative *Staphylococcus* without previous antibiotic treatment had better susceptibility to vancomycin and LVFX (Ta et al., 2003).

Contact lenses are widely used for vision correction, enhancing corneal healing, and drug delivery. Orthokeratology lens (OKL) wearers have a lower abundance of Bacillus, Tatumella, and Lactobacillus, while the abundance of Delftia decreases in soft contact lens wearers when compared with subjects who do not wear contact lenses (Zhang et al., 2017). Interestingly, lens wearers appear to have ocular flora more like skin flora with higher abundances of Methylobacterium, Lactobacillus, Acinetobacter, and Pseudomonas (Shin et al., 2016).

Eye drops are a major drug delivery route for ophthalmic disease. A significantly lower ocular surface-positive culture rate in the treatment group with glaucoma eye drop usage was observed than in the control group without any treatment (Honda et al., 2011). Preservatives commonly used in eye drops have aroused attention. Travoprost and latanoprost are eye drops for glaucoma, but they both use a different preservative agent: the former is SofZia-preserved, and the latter is benzalkonium chloride-preserved. Bacterial samples were collected from conjunctiva sacs of patients who have been treated with travoprost or latanoprost for more than 1 year. The analysis showed that S. epidermidis present in the latanoprost group was significantly more resistant to LVFX, gatifloxacin, moxifloxacin, and tobramycin (Ohtani et al., 2017).

Trauma is an influential factor of OSM. The abundance of *Pseudomonas fluorescens* and *Pseudomonas aeruginosa* is significantly elevated in patients following a traumatic corneal ulcer when compared with healthy subjects (Kang et al., 2020). Dry eye disease (DED) occurs when tears cannot provide adequate lubrication and nourishment to the ocular surface. It is classified into two subtypes: aqueous tear-deficient dry eye (ADDE) and hyper-evaporative DED (Craig et al., 2017). Compared with the normal healthy population, there is significantly lower diversity and relative abundance of OSM in patients with ADDE (Andersson et al., 2021). Another study has compared the difference in OSM between DED and non-DED subjects, revealing depleted levels of *Pseudomonas plecoglossicida, Pseudomonadaceae, Gammaproteobacteria*, and *Proteobacteria* in DED eyes (Li et al., 2019). Further reports have indicated that lifestyle and systemic disease may alter OSM. For example, OSM in chronic alcoholism shows a significantly higher presence of Staphylococcus aureus (Gunduz et al., 2016). In patients with diabetes mellitus, Proteobacteria makes up a large proportion of OSM, whereas Firmicutes is the most common phylum of the OSM in healthy populations (Ham et al., 2018).

#### Acquired factors

Conjunctiva is a low-biomass tissue. Even a little DNA contamination can produce false-positive signals, especially after PCR amplification, so a rigorous contaminant-filtering step is needed to eliminate the “noise” from the environment overwhelming the target signal (Glassing et al., 2016). Moreover, it is hard to distinguish the bacteria that are resident on the ocular surface or accidentally brought there by finger, contact lens, or wind. Within the microbiota community, complicated interactions, including competition and cooperation, happen simultaneously and reach a state of dynamic equilibrium; therefore, the OSM profile may differ at every sampling time point (Wintermute and Silver, 2010; Kang et al., 2021). The sampling site also contributes to this variation as the distribution of OSM is inconsistent (Ozkan et al., 2019). In addition, it is crucial to distinguish whether the origin of the recovered samples is from viable or nonviable organisms. A harvested sample may be simply microbes perishing in the antimicrobial microenvironment, instead of a community of colonizing organisms (Zegans and Van Gelder, 2014). Also, the baseline state may be complicated by diet, age, and environment, as previously mentioned. Further research is needed to enhance previous findings by providing a much more detailed examination.

## Relationship between OSM, immunity, and ophthalmic infectious disease

### Crosstalk between OSM and innate immunity

OSM is reported to boost the local innate immunity, link it to adaptive immunity, and maintain a harmonious relationship between them, pathogenic microbials, and the host.

*Corynebacterium mastitidis* is a commensal organism residing on the ocular surface of humans and mice. In a mouse model, it triggers γδ T cells within the ocular mucosa to produce IL-17. IL-17 then activates downstream synthesis of antimicrobial substance into tears to strengthen protection from invasive *Candida albicans* and *Pseudomonas aeruginosa*, expounding the indispensable role of microbiota–T-cell interaction in ocular surface innate immunity (St Leger et al., 2017). A lack of bacterial colonization may weaken the corneal barrier as significantly greater corneal barrier disruption and more goblet cell loss were found within the lacrimal gland of the germ-free (GF) mice than in the conventional C57BL/6J mice (Wang et al., 2018).

Secretory IgA (SIgA), the predominant antibody in tears and the ocular surface, is a first-line defensive system in the ocular surface (Knop et al., 2008). In GF mice, protein and heavy-chain transcription of SIgA are significantly decreased on the ocular surface when compared with conventional mice. Similarly, both wild-type mice treated with ophthalmic gentamicin gel and GF mice show reduced gene expression of the downstream effectors of IL-17 (St Leger et al., 2017). Complement component 3 (C3) and C9 are important innate immune effectors, which decrease in eyewashes of GF mice compared with specific pathogen-free Swiss Webster mice (SPF SW mice) (Kugadas et al., 2016). These findings strongly suggest that OSM participates in ocular surface innate immunity to a great extent as either temporal interference in OSM *via* antibiotics or artificial depletion of OSM reduces the integrity of the ocular mucosal barrier and innate immunity.

### Crosstalk between OSM and adaptive immunity

Interlukin-1 beta (IL-1ß) is a member of the interlukin-1 family. It connects innate immunity and adaptive immunity as it shows to increase nucleoprotein-specific CD4+ and CD8+ T-cell immunity (Van Den Eeckhout et al., 2021). It may serve as a possible driving adjuvant in Th-17 cell-mediated immune response (Wüthrich et al., 2013), and it is necessary for antigen-specific T-cell activation (Nambu et al., 2006). Kugadas et al. found significantly lower expression of corneal IL-1ß in *P.aeruginosa* PAO1-infected tissues from mice treated with local gentamycin. This illustrates that the existence of OSM could promote the release of IL-1ß during ocular infection (Kugadas et al., 2016).

Sjogren syndrome (SS) is an autoimmune disease attacking the exocrine glands. It leads to severe dryness of the mucosal surfaces. CD4+ T cells are involved in SS; overactive CD4+ T cells create glandular injury *via* the activation of B cells (Singh and Cohen, 2012; Brito-Zerón et al., 2016). Zaheer et al. compared CD25 knockout (CD25KO) mice, which spontaneously developed SS-like inflammation, in GF and conventional conditions. GF CD25KO mice had a higher total lymphocyte infiltration score, greater expression of IFN-γ and IL-12, and a higher frequency of autoreactive CD4+ T cells than conventional CD25KO mice. However, fecal transplantation from C57BL/6J mice reversed the phenotype of dry eyes and lowered the generation of pathogenetic CD4+ T cells in GF CD25KO mice. This study highlights that a lack of microbiota may accelerate the production of CD4+ T cells with greater pathogenicity and the immunomodulatory properties of microbiota. Furthermore, it implies that the microbiota in the gut may affect the ocular condition of its host (Zaheer et al., 2018).

In summary, OSM is involved in the release of antimicrobial molecular and signals, SIgA production, the regulation and promotion of the immune system, and the connection between native immunity and adaptive immunity.

### Ophthalmic infectious disease and OSM

The knowledge of the characteristics of OSM in innate immunity and adaptive immunity has led to studies addressing the relationship between altered OSM and ophthalmic infectious disease.

Blepharitis is general inflammation along the edges of the eyelids (Lindsley et al., 2012). Increased relative abundances of *Staphylococcus, Streptophyta, Corynebacterium*, and *Enhydrobacter* were noted in the eyelash and tear samples from subjects with blepharitis, suggesting blepharitis may be caused by exposure to pollens, dusts, and soil particles (Lee et al., 2012). Previous reports indicated that an elevated proportion of skin microflora on the ocular surface may lead to blepharitis. This corresponds to Lee et al. (2012) as they found a significantly higher Staphylococcus proportion in subjects with blepharitis (Groden et al., 1991; Kulaçplu, 2001).

Dysbiosis in the ocular fungal microbiota is related to fungal keratitis (FK). Prashanthi et al. collected conjunctival swabs (SWs) from a healthy control (HC) group and additional corneal scrapings (CRs) from participants with FK. A significant difference in the Shannon index, Simpson index, and number of observed OTUs were noted between HC-SW and FK-CR groups. Ascomycota (mean abundance, 35.66%) and Basidiomycota (mean abundance, 37.05%) were the two most dominant phyla in the HC-SW. Ascomycota was the most dominant phyla in the FK-CR and FK-SW; however, the abundance was clearly increased (mean abundance was 88.36% and 69.99% in FK-CR and FK-SW, respectively). *Basidiomycota* was significantly lower in both FK-CR and FK-SW groups; it accounted for only ≤10.2%. Interaction networks were established based on pair-wise correlations. “Hub genera” refers to a genus interacting with more than 10 genera in the network, either positively or negatively. In HC-SW, *Setosphaeria* was the largest hub genera, which negatively interacted with 20 other genera, symbolizing its inhibitive role in the growth of *Pseudozyma, Peniophora, Trichomonascus*, and *Talaromyces* (Prashanthi et al., 2019).

Trachoma, caused by Chlamydia trachomatis, is the most common infectious cause of blindness. It is characterized by conjunctival inflammation, scarring, trichiasis, or entropion (Taylor et al., 2014). Zhou et al. recruited 210 residents in Gambia, 105 with healthy conjunctivae and 105 with clinical signs of trachoma; decreased diversity and increased abundance of Corynebacterium and Streptococcus were noted in the participants with signs of trachoma (Zhou et al., 2014).

Aforementioned studies explained the close connection between ophthalmic infectious disease and OSM; however, the interest lies in whether alterations or improvements in OSM can serve as a treatment strategy in ophthalmic infectious disease. Kugadas et al. indicated that GF mice are more susceptible to *Pseudomonas aeruginosa*-induced keratitis. This may be caused by commensal microbiota deficiency. Coagulase-negative *Staphylococcus sp.*, the predominant bacterium in OSM, was topically applied to the ocular surface of GF mice. After 2 weeks, the mono-colonized GF mice showed restoration of resistance to P. aeruginosa and the same expression level of IL-1ß conjunctival transcription as Swiss Webster mice. The restoration of resistance toward pathogens and OSM homeostasis *via* topical application of commensal bacteria is an inspiring finding (Kugadas et al., 2016). In humans, topical or systemic administration of specific commensal bacteria to treat ophthalmic infectious disease has gathered great importance.

## The gut–eye axis and potential of probiotics in treating ophthalmic infectious disease

Human gut microbiota (HGM) comprises more than 100 trillion microorganisms and can weigh up to 2 kg in an adult, which has coevolved in eclectic physiological responses, including digestion, metabolism of xenobiotics, antimicrobial peptide production, and immunomodulation by upregulation or downregulation of host gene expression (Fu et al., 2017). Up to 116 genes are significantly differentially expressed in GF mice and conventional mice (Nichols and Davenport, 2021). Components of HGM differ from person to person and can be influenced by diet, lifestyle, and medication treatment. Currently, microbiota colonized out of eyes, particularly in the gastrointestinal tract, are found to be related to several ophthalmic diseases, and such linkage is the so-called gut–eye axis (Clemente et al., 2012; Turner, 2018; Napolitano et al., 2021).

Astafurov et al. compared the oral cavity microbiota of a healthy population and that of patients with glaucoma *via* mouthwash specimens. Significantly higher bacterial loads are found in glaucoma cases, and linear discriminant analysis of DNA has confirmed differences in oral cavity microbiota of the two groups (Astafurov et al., 2014). HGM also participates in the modulation of age-related macular degeneration (AMD). Serotonin (5-HT) is a protective substance against retinal damage, and its synthesis is mainly regulated by gut spore-forming bacteria (Yano Jessica et al., 2015; Zhang and Davies, 2016). Corroborating outcomes are noted in another study, indicating that modifying HGM with a low-glycemia diet can delay the onset and progress of AMD features in animal models (Rowan et al., 2017).

Ophthalmic infectious disease is related to HGM. An increased abundance of the pathogenic fungi, *Aspergillus* and *Malassezia*, is noted in fecal samples collected from patients with bacterial keratitis (BK). The Shannon and Simpson indexes of HGM are significantly different between the BK and HC groups (Jayasudha et al., 2018). SPF SW mice are more susceptible to ocular *P. aeruginosa* infection and have increased corneal pathology with depletion of gut microbiota (Kugadas et al., 2016). In addition, a decline in SIgA expression in the tears has been noted in conventional SW mice upon oral antibiotic treatment (Kugadas and Gadjeva, 2016). These studies are indicative of the fact that the interference in gut microbiota may lead to decreased ocular immunity. It should not be neglected that the discrepancy between the HGM of the normal healthy population and that of patients with infectious ophthalmic disease was never limited in taxonomic abundance; functional differences in HGM between HC and FK patients were reported. A total of three KEGG pathways are significantly enriched in the FK group. Another six KEGG pathways are enriched in the HC group, including the biosynthesis of ansamycin and neomycin (Kalyana Chakravarthy et al., 2018).

The overuse of antibiotics has led the emergence of multidrug-resistant (MDR) bacteria; multidrug-resistant *Pseudomonas aeruginosa* (MDR-PA) and methicillin-resistant *Staphylococcus aureus* (MRSA) have been reported in ophthalmic infectious disease (Vazirani et al., 2015; Bispo et al., 2020). Therefore, an alternative therapy to antibiotics is required to overcome this issue. Numerous studies provide further demonstration by emphasizing the relationship between alterations in HGM and ophthalmic health. This clearly supports the collaborative contribution between OSM and HGM in the homeostasis of eyes. Novel therapies based on these findings have been studied in recent years. “Probiotics” are live nonpathogenic microorganisms administered to improve microbial balance, particularly in the gastrointestinal tract, which beneficially affects the host (Schrezenmeir and Vrese, 2001; Williams, 2010). They are expected to serve as brand new therapies for ophthalmic infectious disease *via* improving the HGM and adjusting the microenvironment in human eyes, thus ameliorating ophthalmic infection. A study has screened over 2,000 strains to select the bacterial strains that met 12 criteria including having specific biochemical activity, withstanding to low-pH environments, and exerting a beneficial impact on the host. Finally, two commercial probiotic strains, *Lactobacillus rhamnosus* GG and *Lactobacillus acidophilus* LA-1, were picked up. Following this, several strains belonging to *Lactobacillus* and *Bifidobacterium* have been confirmed as probiotics (Prasad et al., 1998; Picard et al., 2005; Lebeer et al., 2008).

## Antimicrobial effect of probiotics to pathogens in ophthalmic infection

Probiotics can affect pathogens, as shown by current studies in the field. These highlight the antimicrobial capacity of probiotics toward pathogens that cause ophthalmic infection.

Biofilms are clusters of microorganisms that stick on the surface of plants or animals, being encased in an outer polymer layer that can be produced by microorganisms and able to evade the immune response or resist antibiotics (Hall-Stoodley et al., 2004). *Bacillus spp*. can form biofilm on the ocular surface. This was the target of Akova et al. They tested eight species of probiotics belonging to Lactobacillus for their anti-biofilm capacity; five of them significantly lowered biofilm formation compared with the control group (Akova et al., 2021).

A total of 17 culture-positive (*Staphylococcus aureus* in four isolates and *S. epidermidis* in 13 isolates) swabs were collected from patients with bacterial conjunctivitis. Antibiotic susceptibility profiling was performed, and all *Staphylococcus spp*. were resistant to oxacillin, penicillin G, and cephalexin. In total, six strains of probiotics belonging to *Lactobacillus* and *Bifidobacterium* were prepared to evaluate their antimicrobial potential. Interestingly, all the probiotics in this study expressed promising inhibition on bacterial growth, even on species with antibiotic resistance (Mohamed et al., 2020). Gonococcal conjunctivitis in neonates is caused by Neisseria gonorrhoeae colonizing in the maternal birth canal, which may result in severe vision impairment or blindness (Mallika et al., 2008). Biogenic substance of *L. rhamnosus* strain L60 could suppress the growth of *Neisseria gonorrhoeae* (Ruíz et al., 2015). Another pathogen of neonatal conjunctivitis is herpes simplex virus 2 (HSV-2), which infects neonates during vaginal birth (Brown et al., 2007). In a mammalian cell model, *Lactobacillus crispatus* prevented HSV-2 entry into cells by trapping the viral particles and blocking HSV-2 receptors (Mousavi et al., 2018).

Parasite infection also threatens human vision. Toxocariasis, caused by *Toxocara canis* infection, is a common zoonotic disease that has ophthalmic involvement, known as ocular larva migrans (Aydenizöz-Özkayhan et al., 2008; Chen et al., 2018). Traditionally, benzimidazole and steroids are the only remedy (Magnaval et al., 2001). However, a lower *Toxocara canis* burden was found in mice inoculated with *Enterococcus faecalis* CECT 7121 before infection compared with mice without inoculation (Basualdo et al., 2007).

A possible mechanism for these reports includes nutrition competition, immune modulation, or production of inhibitory substances reducing pathogen activity. Taken together, probiotics may become a new therapy for ocular toxocariasis.

## Current application of probiotics in ophthalmic disease and limitations

Probiotics may be a promising therapy in future; however, several challenges are to be faced. Probiotic administration occurs *via* oral intake, fecal transplantation, and topical use. The first two are based on the gut–eye axis; expecting alterations in HGM may have positive or curative effects on ophthalmic disease. The final route is a more intuitive concept that directly changes OSM and modifies the ocular microenvironment.

The inhibition of pathogens can be caused by bacteriocin, ribosomally synthesized peptides, or proteins with antimicrobial activity produced by probiotics (Gálvez et al., 2007). Lactobacillus strains suppress bacterial growth by lowering the pH of their incubation medium with acetic and lactic acids (Fayol-Messaoudi et al., 2005). Commensal bacteria can inhibit pathogen growth through competition for nutrition and space (Miller and Iovieno, 2009; Kang et al., 2021). However, neither bacteriocin nor organic acids have been reported to approach eyes via systemic circulation or other routes. A previous study has shown higher expression of ansamycin and neomycin biosynthesis in healthy subjects than in patients with FK (Kalyana Chakravarthy et al., 2018). Nevertheless, whether these pathways are the key point in the inhibition of pathogen growth is unclear. Competition between commensal bacteria and pathogens may take place in adjacent areas alone. This is unlikely to suppress the growth of ocular pathogens directly from a distant organ. The oral administration or fecal transplantation of probiotics as new therapies for ophthalmic infectious disease treatment seems uncertain, and more research is required to pursue this line of investigation. For topical administration, probiotic eye drops have been developed for use in vernal keratoconjunctivitis (Iovieno et al., 2008) but not for infectious diseases.

In the field of utilizing probiotics as a new therapy for eye infection, much remains to be done. First, it is essential to investigate the precise mechanism and interaction between different probiotics and pathogens. Next, the “antimicrobial spectrum” of each strain should be established, for making it possible to select the most suitable probiotic strain depending on different pathogens. A key area for future research will be the comparison of the efficacy, clinical outcome, and cost-to-performance ratio between probiotics and existing antibiotics, and antiviral agents. Second, the positive effect of probiotics may only be asserted with a high number of viable cells reaching the ocular surface. Ameliorated manufacturing processes and storage ways are necessary as many probiotic bacteria may die during the manufacture and storage of products (Kailasapathy and Chin, 2000; Evivie et al., 2017). Third, the ocular surface contains a wide array of defense mechanisms against microorganisms. It is not clear whether probiotic bacteria can survive persistently. In addition, the ocular surface is exposed to the environment; therefore, numerous internal and external factors, including personal habits, contact lens wearing, eye rubbing, artificial tear usage, and systemic diseases, can alter its microenvironment and OSM state and then influence probiotic bacteria survival (Petrillo et al., 2020). Therefore, it is difficult to estimate the outcome of topically administered probiotic treatment for ophthalmic infectious disease. Last, although probiotics exert satisfying antimicrobial activities on different pathogens, the results are rarely confirmed in human clinical trials. More studies are urgently needed to confirm the efficacy and safety in human beings.

## Conclusions and futural directions

With new sequencing technology, investigations of OSM are no longer limited to culture-dependent methods. The frequent fluctuation of ocular surface microenvironments and highly varied OSM per person mean that the existence of a core microbiota lacks empirical support. However, OSM modulates the immune system, inhibits microbial growth, and plays a crucial role in maintaining homeostasis. Different patterns in OSM and HGM have been reported in different ophthalmic infectious diseases; however, whether the alteration of microbiota is a cause or an outcome of infection is yet to be elucidated. Furthermore, some of current studies, despite being encouraging, did not utilize a filter when investigating OSM. Therefore, amelioration in methodology with a more rigorous or standardized filter is needed to exclude potential contaminations and make the studies more comparable. Futural studies should be alerted to. While research on these novel therapies is still at an early stage, probiotic application in ophthalmic infectious disease is a promising line of inquiry.

## Author contributions

M-CC and EC contributed to the design and implementation of the research and the writing of the manuscript. All authors contributed equally and approved the final version of the manuscript.

## Funding

This study was supported by grants from the Ministry of Science and Technology (MOST), Taiwan (MOST 109-2320-B- 002-051-MY2).

## Conflict of interest

The authors declare that the research was conducted in the absence of any commercial or financial relationships that could be construed as a potential conflict of interest.

## Publisher's note

All claims expressed in this article are solely those of the authors and do not necessarily represent those of their affiliated organizations, or those of the publisher, the editors and the reviewers. Any product that may be evaluated in this article, or claim that may be made by its manufacturer, is not guaranteed or endorsed by the publisher.
